# Editorial: Women in neurotrauma 2023

**DOI:** 10.3389/fneur.2024.1532321

**Published:** 2024-12-10

**Authors:** Laura Lippa, Elham Rostami, Alba Scerrati, Marina Zeldovich

**Affiliations:** ^1^Department of Neurosurgery, ASST Ospedale Niguarda, Milan, Italy; ^2^Department of Neuroscience, Karolinska Institutet (KI), Stockholm, Sweden; ^3^Department of Medical Sciences, Section of Neurosurgery, Uppsala University, Uppsala, Sweden; ^4^Department of Translational Medicine, University of Ferrara, Ferrara, Italy; ^5^Faculty of Psychotherapy Science, Sigmund Freud University, Vienna, Austria; ^6^Institute of Psychology, University of Innsbruck, Innsbruck, Austria

**Keywords:** neurotrauma and neurodegenerative disease, pediatric neurotrauma, traumatic brain injury, women in neuroscience, neuroimaging, public health care, diversity in academia

## Introduction

*Women in neurotrauma 2023* covers a wide range of areas in neurotrauma research, highlighting the intersection of gender, social determinants of health, and advances in neuroimaging and treatment approaches. The research included focuses primarily on adult and pediatric traumatic brain injury (TBI), but also touches on other neurotrauma-related conditions such as dementia. Furthermore, the goal of this Research Topic was to engage female scientists and clinicians in an effort to reduce gender bias in neurotrauma research.

TBI represents a significant portion of the global injury burden and is a leading cause of morbidity, disability, and mortality across all ages (1). Survivors often face lifelong challenges affecting social relationships, quality of life, work capacity, and societal participation (2). Despite its widespread impact, TBI is often considered a hidden disability, as its consequences are frequently cognitive and psychiatric rather than physical. A major challenge in developing effective treatments, both acutely and in long-term rehabilitation, lies in the heterogeneity of TBI, with varied causes, pathology, patient demographics, and outcomes. While anyone can sustain a TBI, certain groups, like victims of intimate partner violence (IPV), are at higher risk. It is estimated that up to 75% of IPV victims suffer a TBI, often from blows to the head or strangulation (3).

The purpose of this editorial is to summarize and categorize research into: (1) adult neurotrauma, (2) pediatric neurotrauma, (3) advances in neuroimaging, and (4) diversity and public health in neurotrauma care. These topics represent the breadth of research and provide a comprehensive overview of dimensions of neurotrauma. The variety of study types, including systematic or narrative reviews, original research studies, or case reports, further reflects the multifaceted nature of this research area.

## Neurotrauma in adults

Rojczyk et al. examined the impact of neuropsychiatric conditions such as posttraumatic stress disorder, depression, and substance use disorders on the odds of intimated partner violence (IPV) perpetration among male veterans of the Iraq and Afghanistan wars diagnosed with mild TBI (mTBI). Results indicate that veterans with mTBI and psychiatric disorders are significantly more likely to engage in IPV, with aggression associated with microstructural changes in the limbic system, particularly the amygdala-hippocampal complex. This suggests that neurotrauma-related brain abnormalities may contribute to violent behavior and calls for targeted interventions in this vulnerable population.

Molero et al. examined medication use patterns in patients after TBI. The study employed a population-based matched cohort in Sweden to assess prescription rates of non-psychotropic medications before and after injury. Results suggest that TBI patients are more likely to use a wide range of medications compared to a control cohort, with significant use of non-steroidal anti-inflammatory drugs and antibiotics. Furthermore, older patients and women tend to have higher rates of medication use, raising concerns about polypharmacy and its implications for post-TBI care.

## Pediatric neurotrauma

Parsons et al. investigated the influence of social determinants of health (SDH) including race, insurance status, and neighborhood opportunity on outcomes after pediatric TBI. The study revealed that Black and Hispanic children are more likely to suffer from severe TBI and experience higher social vulnerability compared to White children. Although no significant differences were observed in mortality or functional outcomes across racial groups, the study highlighted the complex role of SDH in pediatric TBI, suggesting that further research is needed to explore these interactions and their long-term impacts on recovery.

Zeldovich et al. conducted a psychometric evaluation of the German Post-concussion Symptom Inventory (PCSI-SR8) for children aged 8–12 years. The study aimed to validate this self-report instrument for the assessment of post-concussion symptoms in pediatric populations. The research suggests that the inventory reliably captures cognitive, emotional, and physical symptoms associated with pediatric TBI, allowing clinicians to more accurately assess symptom burden. Reference values are provided to facilitate more effective comparisons between TBI patients and children from the general population in future clinical and research settings.

## Advancements in neuroimaging

In a narrative review, Kadaba Sridhar et al. investigated the use of structural neuroimaging markers to differentiate normal pressure hydrocephalus (NPH) from Alzheimer's disease (AD), Parkinson's disease (PD), and the effects of chronic traumatic brain injury (cTBI). Given that NPH is a treatable cause of dementia, distinguishing it from AD and PD, which cause irreversible cognitive decline, is crucial. The study reviewed various neuroimaging markers, particularly from MRI studies, and emphasized the need for computational methods to improve the non-invasive diagnosis of these conditions, which will help increase the accuracy of diagnostic techniques aiming to improve treatment outcomes for patients with neurodegenerative diseases.

Harlyjoy et al. presented a case report of a rare life-threatening complication of TBI called traumatic tension pneumocephalus. The study, based in Indonesia, highlighted the barriers to timely management, including patient refusal of surgery and financial constraints. Despite these challenges, the patient eventually underwent emergency neurosurgery and achieved a full recovery. This case underscores the importance of overcoming healthcare access barriers in low- and middle-income countries to improve neurotrauma outcomes.

## Diversity and public health in neurotrauma care

In a perspective article, Churchill et al. addressed the persistent gender and racial disparities in leadership, academic publishing, and clinical trial management in the field of neurotrauma. The article highlighted the underrepresentation of women and racial minorities in leadership positions, noting that women are less likely to be invited to publish or serve as principal investigators in clinical trials. The authors called for systemic changes to promote diversity and inclusion, emphasizing that progress in neurotrauma research and practice requires the active participation of underrepresented groups.

Ramsay et al. explored the practices of brain injury physicians regarding firearm injury prevention counseling for TBI patients. Given the elevated risk of suicide and firearm-related injuries in this population, the study found that while most physicians believed in the importance of counseling, only a minority routinely discussed firearm safety with their patients. Moreover, physicians who had received formal training on the Research Topic were significantly more likely to inquire about patients' access to firearms, underscoring the need for better education and training in this area to improve safety outcomes for TBI patients.

## Conclusion

*Women in neurotrauma 2023* presents a multifaceted view of neurotrauma research, ranging from clinical advances in neuroimaging to the influence of social determinants of health on pediatric and adult neurotrauma outcomes. Key contributions include the identification of risk factors for IPV perpetration among veterans, advances in medication management after TBI, and the development of diagnostic tools for neurodegenerative diseases. The Research Topic emphasizes the importance of addressing gender and racial disparities and advocates for systemic changes to promote diversity in neurotrauma leadership and clinical trials.

## References

[1] James SL Theadom A Ellenbogen RG Bannick MS Montjoy-Venning W Lucchesi LR. Global, regional, and national burden of traumatic brain injury and spinal cord injury, 1990–2016: a systematic analysis for the Global Burden of Disease Study 2016. Lancet Neurol. (2019) 18:56–87. 10.1016/S1474-4422(18)30415-0 30497965 PMC6291456

[2] Majdan M Plancikova D Brazinova A Rusnak M Nieboer D Feigin V. Epidemiology of traumatic brain injuries in Europe: a cross-sectional analysis. Lancet Publ Health. (2016) 1:e76–83. 10.1016/S2468-2667(16)30017-2 29253420

[3] Colantonio A Valera EM. Brain injury and intimate partner violence. J Head Trauma Rehabil. (2022) 37:2–4. 10.1097/HTR.0000000000000763 34985028 PMC8890689

